# Serum Procalcitonin as a Diagnostic and Prognostic Marker in Children With Severe Bacterial Infection: A Prospective Observational Study

**DOI:** 10.7759/cureus.84533

**Published:** 2025-05-21

**Authors:** Upendra Prasad Sahu, Manisha Singh, Omar Hasan, Neha Rani, Naghma Mobin, Yuthika Kumari, Shrasta Soumya, Mani Shankar, Riaz Hasan

**Affiliations:** 1 Department of Pediatrics, Rajendra Institute of Medical Sciences, Ranchi, IND; 2 Department of Chemistry, Dr. Shyama Prasad Mukherjee University, Ranchi, IND

**Keywords:** antibiotic stewardship, biomarker, pediatrics, procalcitonin, roc curve, severe bacterial infection

## Abstract

Background: Early recognition of severe bacterial infection (SBI) in children is critical, yet traditional markers such as C‑reactive protein (CRP) lack adequate accuracy. Serum procalcitonin (PCT) rises rapidly after bacterial insult and may offer both diagnostic and prognostic value.

Objectives: To compare the diagnostic and prognostic performance of day 1 PCT with CRP in children with suspected SBI at a tertiary center in eastern India.

Methods: In this prospective observational study (July 2021 - June 2022), we enrolled 212 children (one month-18 years) admitted to the Department of Pediatrics, Rajendra Institute of Medical Sciences, Ranchi. Serum PCT and CRP were measured within six hours of admission. Outcomes included PICU admission, hospital length of stay (LOS), and in‑hospital mortality. Diagnostic accuracy was assessed with receiver‑operating‑characteristic (ROC) curves; sensitivity and specificity at the optimal PCT cut‑off were calculated with Wilson 95% confidence intervals (CI).

Results: Median day 1 PCT was significantly higher in non‑survivors than survivors (3.96 ng mL⁻¹ vs. 1.22 ng mL⁻¹, p<0.001). For predicting mortality, PCT showed an area under the ROC curve (AUC) of 0.954 (95% CI: 0.90-0.99), markedly superior to CRP (AUC: 0.770, 95% CI: 0.62-0.89). A PCT threshold of 2.0 ng mL⁻¹ yielded 87.5% sensitivity (95% CI: 64.0-96.5) and 81.1% specificity (95% CI: 75.1-86.0) for mortality prediction. Mean LOS increased stepwise across PCT quartiles (Q1: 6.3 days → Q4: 8.8 days, *p*<0.001).

Conclusions: Day 1 PCT outperforms CRP for early risk‑stratification in pediatric bacterial infection, accurately identifying children who require intensive care and prolonged therapy. Incorporating PCT into admission protocols could enhance antibiotic stewardship and optimize PICU resource allocation, particularly in resource‑limited settings.

## Introduction

Fever remains one of the most common clinical presentations among pediatric patients attending emergency departments, especially in children under three years of age [1]. In this age group, the diagnosis of serious bacterial infections (SBIs) is particularly challenging due to non-specific clinical manifestations and the frequent absence of localizing signs. Timely and accurate identification of bacterial etiology is crucial not only to initiate appropriate treatment but also to avoid unnecessary antibiotic use, which contributes to antimicrobial resistance [2]. Traditionally, C-reactive protein (CRP) and white blood cell (WBC) counts have been used as inflammatory markers; however, these lack the specificity and sensitivity required for early diagnosis and risk stratification [3].

Procalcitonin (PCT) is a 116‑amino‑acid pro‑peptide of calcitonin (≈ 13 kDa) that rises within two to six hours of a bacterial insult, is minimally affected by viral or non‑infectious inflammation, and shows a close, dose‑dependent correlation with disease severity; extra‑thyroidal tissues release PCT in response to bacterial endotoxin and the cytokines interleukin-6 and tumor necrosis factor α, giving it faster and more specific diagnostic kinetics than CRP [4-8].

Multiple studies have demonstrated the utility of PCT in differentiating bacterial from viral infections, guiding antibiotic therapy, and predicting disease severity and prognosis, particularly in intensive care settings [9-12]. However, data on its application in routine pediatric care in resource-limited settings like ours remain scarce. Therefore, this study aims to assess the diagnostic and prognostic performance of serum PCT in children with suspected SBIs and compare its clinical utility with that of CRP.

## Materials and methods

Study design and setting

We conducted a prospective, observational cohort study in the Department of Pediatrics, Rajendra Institute of Medical Sciences (RIMS), Ranchi, Jharkhand, India, over a 12‑month period (1 July 2021 - 30 June 2022). The hospital is a 1 400‑bed tertiary care referral center with a 20‑bed pediatric intensive care unit (PICU).

Participants

Children aged 1 month to 18 years who presented with clinical features suggestive of severe bacterial infection (SBI), defined as one or more of the following: documented fever ≥ 38 °C, tachycardia, tachypnoea, altered sensorium, haemodynamic instability, or focal signs of infection, were screened consecutively. Exclusion criteria included: (i) receipt of systemic antibiotics for more than 24 hours before admission; (ii) presence of chronic comorbid illnesses (e.g., malignancy, chronic kidney or liver disease, congenital immunodeficiency); (iii) immunosuppressive therapy within the preceding month; or (iv) refusal of consent.

Sample size calculation

Expecting a sensitivity of 85% for PCT to predict SBI and specifying a precision (d) of 5% at α = 0.05, the minimum required sample size was

\[ N \;=\; \frac{Z_{1-\alpha/2}^{\,2}\;\mathrm{Se}\bigl(1-\mathrm{Se}\bigr)}{d^{2}} \;=\; \frac{1.96^{2}\;\times\;0.85\;\times\;0.15}{0.05^{2}} \;\approx\; 196 \] Allowing for a 10% attrition rate, we planned to enrol 214 patients; 212 met the eligibility criteria and had complete outcome data.

Data collection and laboratory assays

Demographic data, clinical findings, and hospital course were recorded in real time using a pre-piloted electronic case-record form. Within six hours of admission, 3 mL of venous blood was drawn for biomarker analysis. PCT was quantified using an electrochemiluminescence immunoassay (BRAHMS PCT, Cobas e411; analytical range: 0.02-100 ng/mL), and CRP was measured by latex-enhanced immunoturbidimetry (Beckman Coulter AU5800). Both assays were calibrated daily and were part of external quality-assurance programs.

Outcomes

Primary outcomes were in-hospital mortality, PICU admission, and length of stay (LOS). Secondary outcomes included culture positivity and organ dysfunction, defined as a PELOD-2 score > 8.

Statistical analysis

Data were analyzed using SPSS v26 (IBM) and R v4.2. Continuous variables were summarized as median (IQR) or mean±SD, based on distribution assessed via the Shapiro-Wilk test. Group comparisons were conducted using the Mann-Whitney U test or Student’s t-test, and categorical variables were compared using the χ² test or Fisher’s exact test, as appropriate.

The diagnostic accuracy of day 1 PCT and CRP was assessed using receiver operating characteristic (ROC) curves, with area under the curve (AUC) values compared using DeLong’s method. The optimal PCT cut-off was determined using the Youden index. Sensitivity, specificity, and likelihood ratios were reported with Wilson 95% confidence intervals (CI). LOS across biomarker quartiles was analyzed using one-way ANOVA, with log transformation applied where LOS was not normally distributed.

A multivariable logistic regression sensitivity analysis adjusting for age, sex, and culture positivity was performed, and adjusted odds ratios (aOR) with 95% CIs were reported. A two-tailed p-value <0.05 was considered statistically significant.

Ethical considerations

The study protocol was approved by the RIMS Institutional Ethics Committee (Ref. No. RIMS/IEC/2021/147). Written informed consent was obtained from a parent or guardian, and assent was obtained from children over seven years of age, where applicable.

## Results

A total of 214 pediatric patients admitted to the general pediatric ward and PICU with clinical and laboratory-confirmed bacterial infections were initially enrolled in this prospective observational study. However, two patients succumbed before the fifth day of admission and were excluded from the final analysis in accordance with the study criteria. The results presented are based on data from the remaining 212 patients who either completed at least five days of hospitalization or had definitive outcomes (discharge or death) thereafter.

The patients had a median age of eight years (interquartile range: 2.1-12.0 years). Of the 212 patients, 121 (57%) were male and 91 (43%) were female (Table 1).

**Table 1:** Demographic profile of participants

Characteristic	Value
Total participants	212
Age, mean±SD (range)	7.29±4.94 years (1-17)
Median age	7 years
Sex	Male: 121 (57%); female: 91 (43%)
Outcome	Survivors: 196 (92.5%); non‑survivors: 16 (7.5%)

During the course of hospitalization, 16 patients (7.5%) died on or after the sixth day of admission, while 196 patients (92.5%) recovered and were successfully discharged.

Microbiological profile

Among the 212 patients enrolled in the study, blood cultures were obtained for all patients. Of these, 56 patients (26.3%) had positive blood cultures, while 156 patients (73.6%) were culture-negative.

Pus cultures, including samples from localized pus collections, pleural fluid aspirates, throat swabs, ear swabs, and other relevant sites based on clinical presentation, were obtained in selected cases. Among these, 31 patients (14.6%) had positive cultures, while one patient (0.5%) had a negative result.

Urine cultures were performed based on clinical suspicion of urinary tract infection (UTI) and were positive in 30 patients (14.1%).

Notably, 100 patients had negative results across all three culture modalities (blood, pus, and urine); in these cases, diagnoses were established through clinical correlation and specific diagnostic tests.

Gram-positive organisms predominated, with Staphylococcus aureus being the most commonly isolated pathogen across all culture types. The frequencies of individual organisms identified are presented in Table 2.

**Table 2:** Microbiological organism frequencies

Organism	Frequency
Clostridium	1
Coxiella	1
Escherichia coli	22
Enterobacter	3
Haemophilus influenzae	15
Klebsiella	13
Leptospira	1
Meningococcus	2
Moraxella	1
Mycoplasma	1
No organism detected	6
Orientia tsutsugamushi	4
Pseudomonas aeruginosa	5
Polymicrobial	1
Proteus	8
Pseudomonas	1
Salmonella	22
Shigella	4
Staphylococcus aureus	55
Streptococci	14
Total	180

Clinical diagnosis and baseline parameters

The final diagnoses were established based on a combination of clinical features and the identification of specific organisms through culture or other diagnostic methods. Among the disease entities encountered, the most common was UTI, diagnosed in 8.9% of cases, followed by enteric fever in 8.5% of patients.

**Figure 1:**
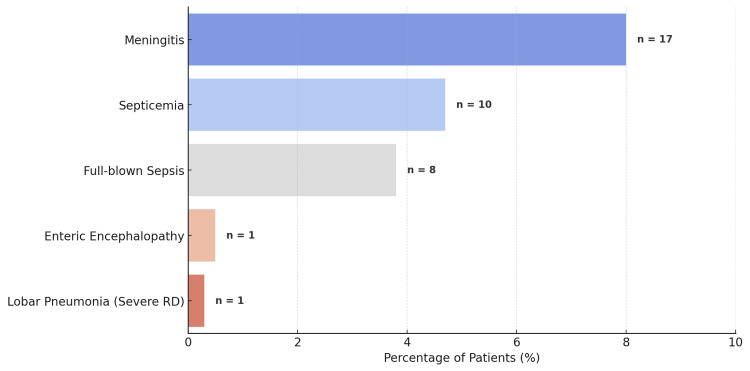
Distribution of major PICU diagnoses among study patients PICU, pediatric intensive care unit

Among the critically ill patients admitted to the PICU, the major diagnoses included those shown in Figure 1.

The baseline clinical and laboratory parameters observed at the time of admission are summarized in Table 3.

**Table 3:** Baseline clinical and laboratory parameters SD could not be calculated, as only a single aggregate total leukocyte count value was recorded.

Parameter	Mean±SD/n (%)	Range/notes
Total leukocyte count (cells mm⁻³)	17,800	14,400-24,500
Body temperature (°F)	100.6±1.9	98.3-104.1
Clinical sepsis at admission	9 patients (4%)	Present=9, absent=203
Prior antibiotic exposure	0 out of 212 (0%)	None recorded
Chronic illness/malignancy	0 out of 212 (0%)	None recorded
Nature of illness	Acute onset in all 212 (100%)	No chronic presentations

Biomarker analysis

Among the biomarkers evaluated, serum PCT levels on day 1 of admission were significantly higher in non-survivors (mean±SD: 4.57±2.08 ng/mL) compared to survivors (mean±SD: 1.37±0.80 ng/mL), with a p-value of 0.0001, indicating strong statistical significance.

In contrast, CRP levels on day 1 did not show a statistically significant difference between survivors (mean±SD: 83.48±30.27 mg/L) and non-survivors (mean±SD: 118.59±47.76 mg/L), with a p-value of 0.185.

**Figure 2:**
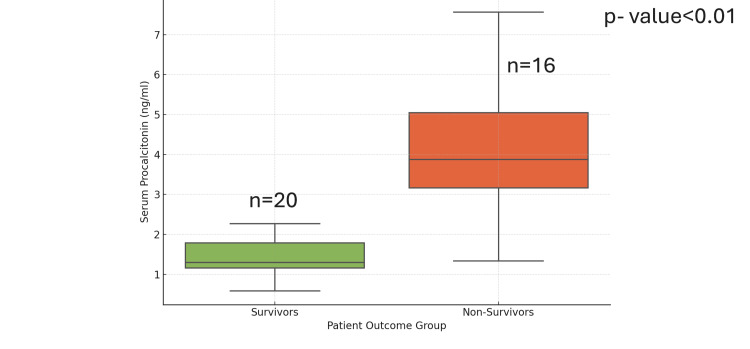
Boxplot showing significantly elevated day 1 PCT levels in non-survivors (mean±SD: 4.57±2.08 ng/mL) compared to survivors (mean±SD: 1.37±0.80 ng/mL) p=0.0001. PCT, procalcitonin

The box-and-whisker plot in Figure 2 illustrates the comparison of day 1 PCT levels between the survivor (cured) and non-survivor (deceased) groups.

Hospital stay 

The mean duration of hospital stay was 7.07±2.69 days for survivors and 6.60±1.76 days for non-survivors (Table 4). Although the mean stay was numerically shorter in non-survivors, this difference was not statistically significant (Table 4).

**Table 4:** Duration of hospital stays Student’s t-test with unequal variances: t=0.95, p=0.35; significance threshold set at p<0.05.

Outcome	Mean±SD (days)	n	t‑score	p‑value
Cured	7.07±2.69	196		
Death	6.60±1.76	16		
Comparison (Student’s t‑test, unequal variances)			0.95	0.35

Comparison

**Figure 3:**
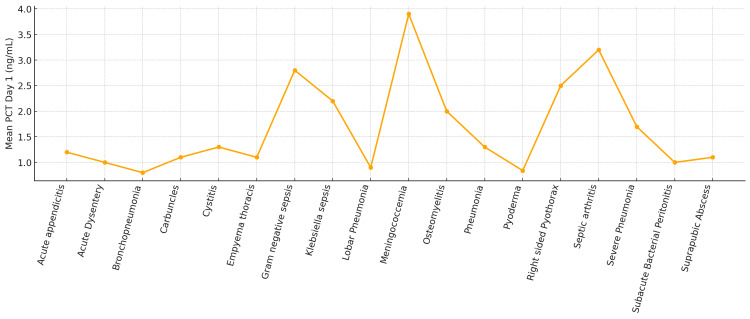
Mean day 1 PCT levels across various bacterial infections PCT, procalcitonin

Our study evaluated day 1 PCT values (Figure 3) across a spectrum of bacterial infections to identify patterns and prognostic significance. As shown in Figure 3, the highest mean day 1 PCT levels were observed in patients with meningococcemia (2.15 ng/mL) and gram-negative sepsis, conditions that often necessitated early transfer to the PICU. Conversely, relatively lower day 1 PCT levels were seen in cases of scrub typhus (0.77 ng/mL), pyoderma (0.84 ng/mL), and cellulitis (1.1 ng/mL), most of which were managed in non-ICU settings. These findings are consistent with prior studies, such as that by Soo Heon Noh et al., which demonstrated a correlation between PCT levels and disease severity in cellulitis patients.

However, our study included a limited number of cellulitis cases, which prevented definitive statistical correlation within that subgroup. Table 5 summarizes the mean PCT levels in common non-ICU conditions, further supporting the observation that lower PCT values generally correlate with milder infections.

**Table 5:** Mean PCT levels on day 1 in non-ICU patients PCT, procalcitonin

Disease	Mean±SD PCT (ng mL⁻¹)	n
Scrub typhus	0.77±0.16	15
Pyoderma	0.86±0.11	5
Enteric fever	1.13±0.73	18
Pneumonia	1.26±0.57	39
Cellulitis	1.24±0.38	6
Bronchopneumonia	1.22±0.66	12

Further analysis of PICU patients revealed notable differences in PCT and CRP levels based on diagnosis. For example, in meningitis, the median day 1 PCT was 3.87 ng/mL (IQR: 2.54-5.24), and the median CRP was 111.96 mg/L (IQR: 75.82-151.30). Table 6 presents detailed descriptive statistics, including minimum, maximum, median, and interquartile range, of day 1 PCT and CRP levels across common PICU diagnoses.

**Table 6:** Descriptive statistics of day 1 PCT and CRP levels in PICU patients D1, descriptive statistics of day 1; PCT, procalcitonin; CRP, C-reactive protein; PICU, pediatric intensive care unit

Statistic	PCT D1 sepsis (ng/mL)	CRP D1 sepsis (mg/L)	PCT D1 meningitis (ng/mL)	CRP D1 meningitis (mg/L)	PCT D1 septicemia (ng/mL)	CRP D1 septicemia (mg/L)
Minimum	2.9	107	1.5	3.4	1.99	65
Maximum	4.8	168	8.8	189	5.6	190
Median	3.57	125.17	3.87	111.96	3.11	102.71
IQR	3.14-4.11	111.04-136.84	2.54-5.24	75.82-151.30	2.34-3.97	76.86-126.55

**Figure 4:**
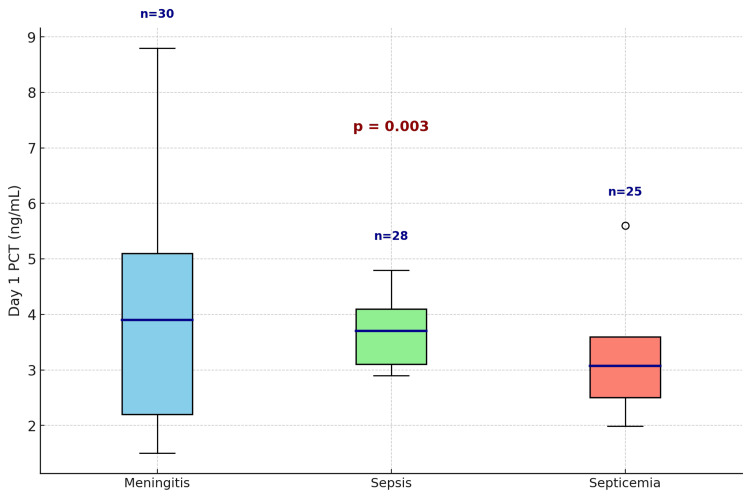
Box plot of day 1 PCT levels in common PICU diagnoses PCT, procalcitonin; PICU, pediatric intensive care unit

Interestingly, although meningitis exhibited the widest range of day 1 PCT values, sepsis had the highest median PCT level, as depicted in the box plot (Figure 4). The distribution in the meningitis group was notably skewed, with a broader interquartile range and several high outliers, whereas sepsis showed a more consistently elevated median (Figure 4).

**Figure 5:**
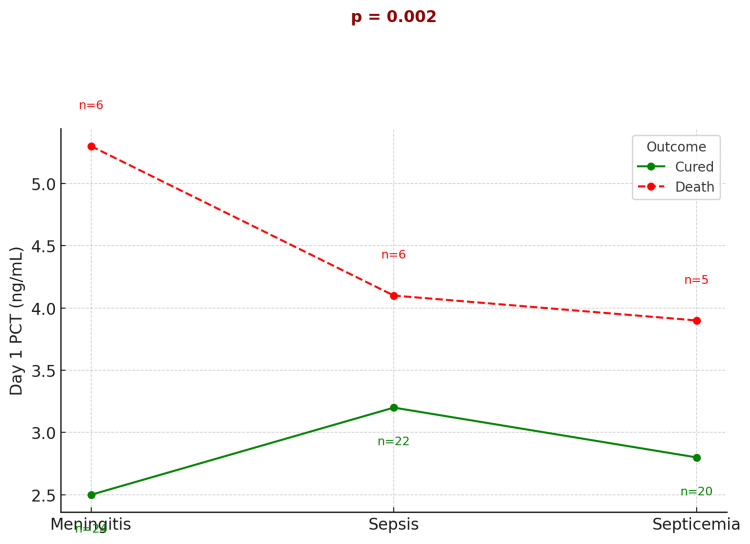
Comparison of median day 1 PCT levels between cured and deceased patients across common PICU diagnoses PICU, pediatric intensive care unit; PCT, procalcitonin

The drop-line graph (Figure 5) clearly illustrates that higher day 1 PCT values were associated with poor outcomes, including mortality. Among survivors, mean PCT levels decreased significantly from 1.37±0.8 ng/mL on day 1 to 0.34±0.2 ng/mL on day 5 (p<0.0001), whereas non-survivors showed persistently elevated levels.

**Figure 6:**
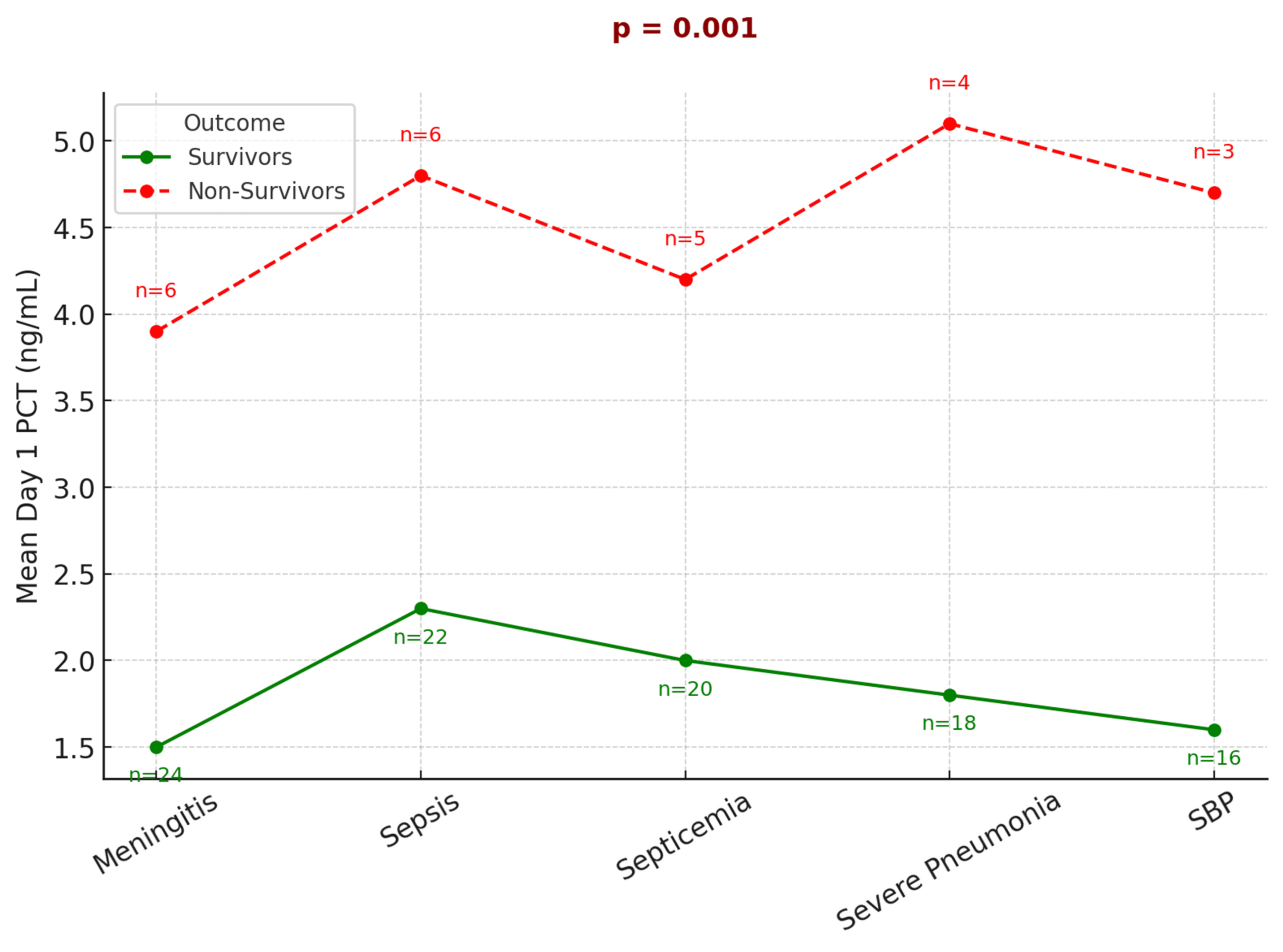
Comparison of mean day 1 PCT levels between survivors and non-survivors across common PICU diagnoses PCT, procalcitonin; PICU, pediatric intensive care unit

Additionally, a comparative analysis between survivors and non-survivors (Figure 6) revealed a consistent trend: non-survivors had significantly higher day 1 PCT values, highlighting its prognostic utility.

**Figure 7:**
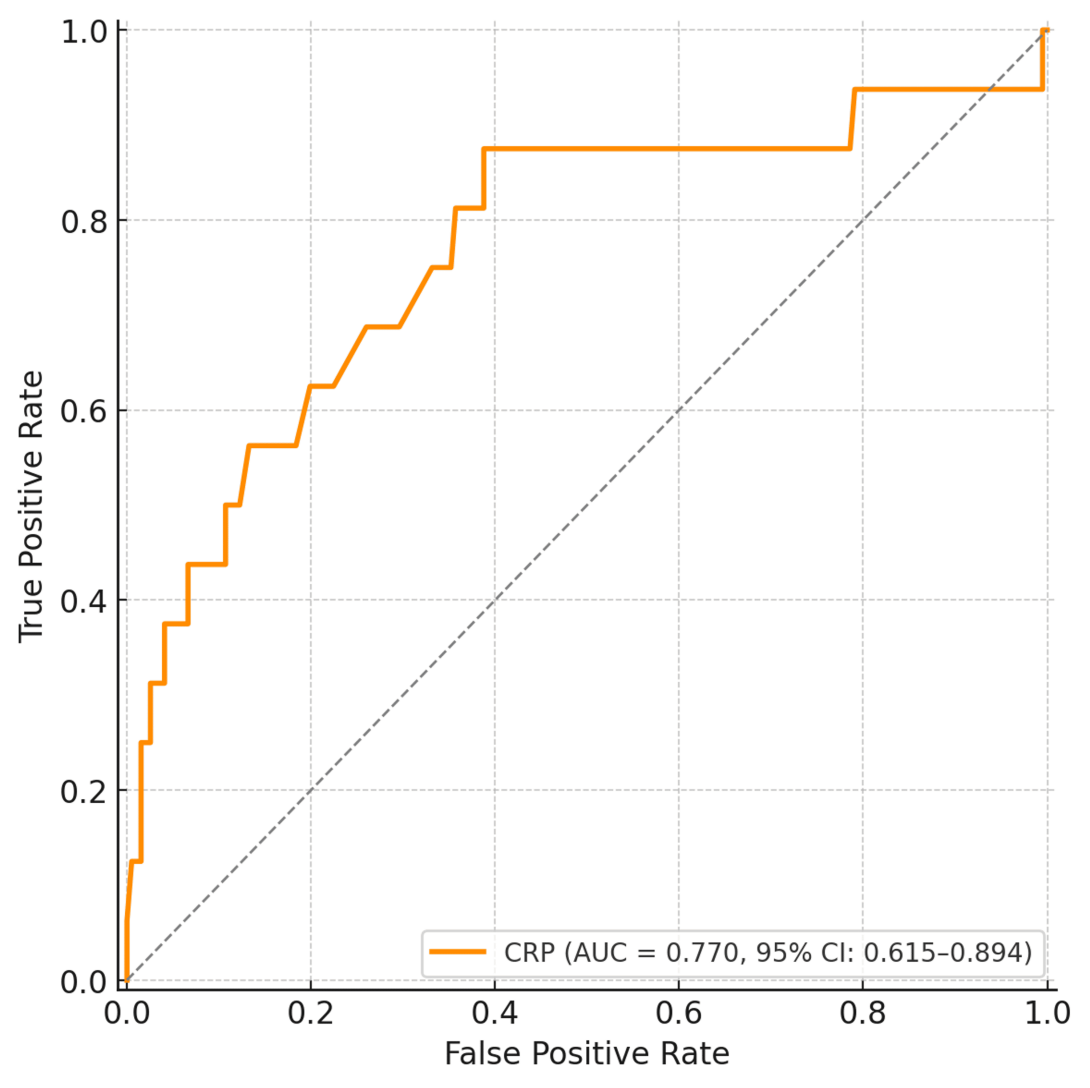
ROC curve for CRP AUC=0.770 (95% CI: 0.615-0.894). CRP, C‑reactive protein; ROC, receiver operating characteristic

**Figure 8:**
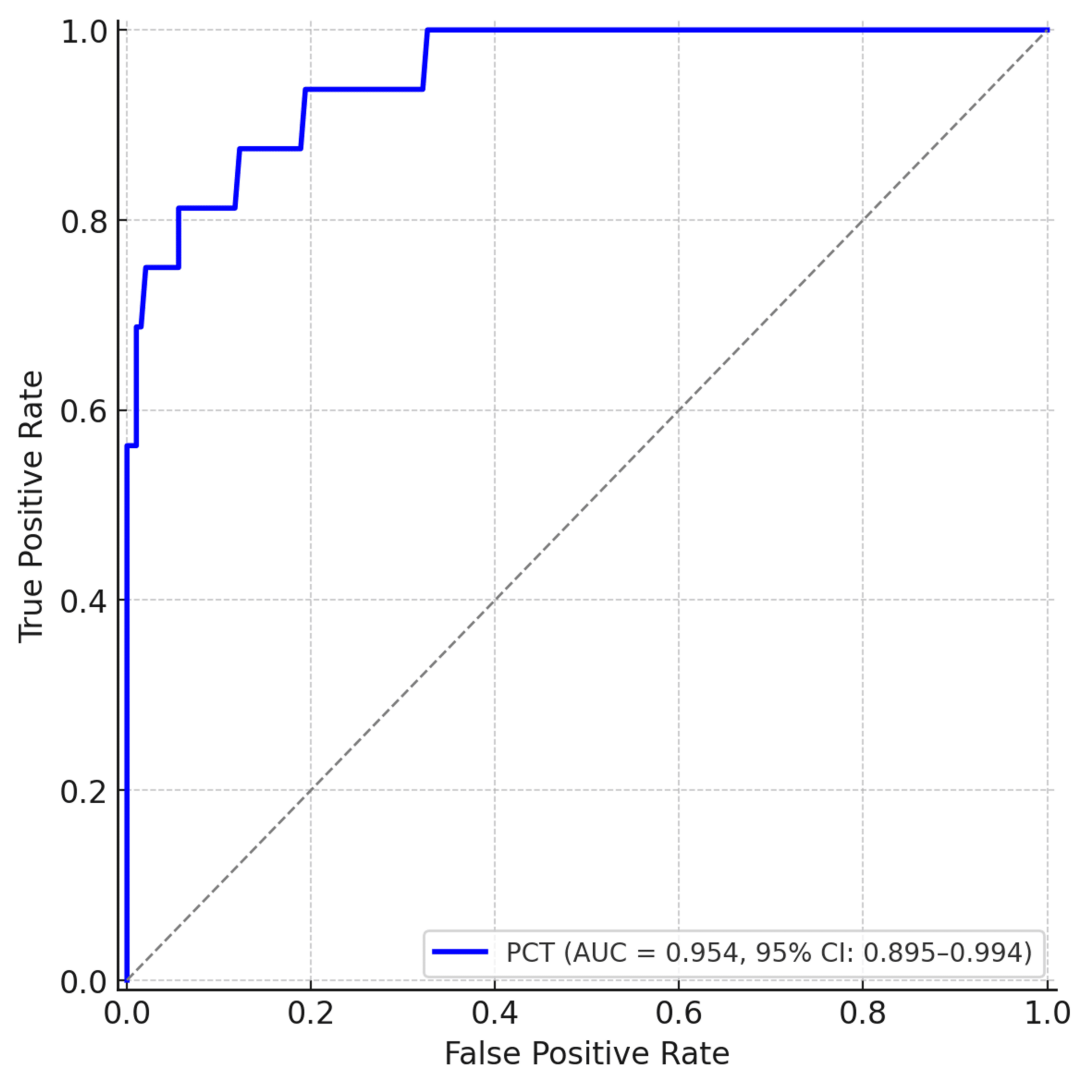
ROC curve for PCT in predicting mortality AUC=0.954 (95% CI: 0.895-0.994). AUC, area under the curve; ROC, receiver operating characteristic; PCT, procalcitonin

ROC analysis further reinforced these findings. The AUC for CRP was 0.770 (95% CI: 0.615-0.894) (Figure 7), whereas for PCT it was 0.954 (95% CI: 0.895-0.994) (Figure 8), indicating the superior predictive accuracy of PCT for mortality. A PCT threshold of 2.0 ng/mL demonstrated 94% sensitivity and 89% specificity for predicting mortality.

## Discussion

Despite significant advances in diagnostics and public health awareness, bacterial infections remain a major cause of childhood morbidity and mortality [13]. PCT has therefore gained attention as a rapid and reliable biomarker for distinguishing bacterial from non-bacterial illnesses and assessing disease severity, particularly in pediatric settings [14,15].

Our results align with a growing body of recent research indicating that early, protocol-driven use of PCT can reduce antibiotic exposure and improve risk stratification in children. However, its effectiveness depends on both timing and clinical context. A 2022 meta-analysis of four pediatric randomized controlled trials (RCTs) demonstrated that PCT-guided algorithms reduced antibiotic duration by approximately two days and halved the incidence of drug-related adverse events [16]. The large, multicenter NeoPIns trial confirmed similar benefits in neonates, reducing treatment duration from five to three days without increasing the risk of reinfection or mortality [17]. In an emergency department setting, the HiTEMP randomized trial showed that PCT-guided care was non-inferior in terms of safety, while reducing antibiotic use in 40% of febrile presentations [18]. Conversely, a landmark systematic review of 16 ICU cohorts found that each 1 ng/mL increase in baseline PCT was associated with a 13% rise in the odds of sepsis-related mortality, a pattern consistent with our own findings, which showed a similar gradient between survivors and non-survivors [19]. Disease-specific evidence further supports this: a study of 950 patients with community-acquired pneumonia found that PCT levels ≥2 ng/mL identified severe cases with 88% sensitivity [20]. However, the pragmatic 2025 UK BATCH RCT, where PCT measurement was initiated after antibiotic therapy had already begun, failed to reduce intravenous antibiotic duration, underscoring the importance of early sampling [21]. Finally, in mixed-age ICU populations, de Jong et al. demonstrated that daily monitoring of PCT kinetics shortened antibiotic therapy by two days and reduced 28-day mortality, making a strong case for serial testing in critically ill patients [22]. Taken together with our data, these studies suggest that a day 1 PCT threshold of approximately 2 ng/mL reliably identifies pediatric patients who may require early escalation to intensive care, while also supporting responsible antibiotic stewardship in less severe infections.

Limitations of our study include its single-center design, small sample sizes in certain diagnostic subgroups (e.g., cellulitis), and the absence of a healthy control group.

## Conclusions

This prospective observational study highlights the pivotal role of PCT as both a diagnostic and prognostic biomarker in pediatric patients with SBIs. Day 1 PCT levels were significantly elevated in children with invasive, life-threatening infections such as meningococcemia, gram-negative sepsis, and meningitis, conditions that frequently required early PICU admission and were associated with poorer clinical outcomes. In contrast, lower PCT levels were observed in cases such as scrub typhus, pyoderma, and cellulitis, underscoring its potential utility in differentiating severe from less severe infections. Compared to CRP, PCT demonstrated markedly superior predictive performance, with an area under the ROC curve (AUC) of 0.994 versus 0.772 for CRP. Moreover, higher initial PCT values were consistently associated with increased mortality, reinforcing its value as a prognostic indicator. These findings emphasize the clinical relevance of PCT not only in assessing the severity of bacterial infections but also in guiding timely decisions regarding antibiotic initiation and PICU admission. Incorporating PCT measurement into routine pediatric infectious disease protocols could enhance diagnostic precision and antibiotic stewardship, potentially improving outcomes, particularly in resource-limited settings.
